# Supplementary material for: GAD65 deficient mice are susceptible to ethanol-induced impairment of motor coordination and facilitation of cerebellar neuronal firing
Source: PLoS One. 2023 May 22;18(5):e0286031. doi: 10.1371/journal.pone.0286031 (PMC10202268; doi:10.1371/journal.pone.0286031)
Supplement: Attachment — Submitted filename: Response to Reviewers 230505_Matsunaga.docx [file pone.0286031.s003.docx]

>>Journal Requirements:

>>Please review your reference list to ensure that it is complete and correct. If you have cited papers that have been retracted, please include the rationale for doing so in the manuscript text, or remove these references and replace them with relevant current references. Any changes to the reference list should be mentioned in the rebuttal letter that accompanies your revised manuscript. If you need to cite a retracted article, indicate the article’s retracted status in the References list and also include a citation and full reference for the retraction notice.

As the Journal Requirements suggested, we have checked our reference list. In this revised manuscript, we have added two articles (new [41] and new [64]) to respond to the Reviewer#1’s comments.

>>6. Review Comments to the Author

>>Reviewer #1: I appreciate the authors' response to my questions. It is interesting to know that with synaptic blockers, the fold change of firing rate in WT and GAD65-KO are similar. Would you mind adding this in the manuscript and discuss the assumptions as in the response? Additionally, I hope the message that the experiments were done in tonic cells only can be emphasized since tonic/no-tonic cells may behave very differently. I appreciate that the authors mentioned this in line 277, but would you mind talking about this more in either method or discussion with Womack and Khodakhah, 2002 J Neurosci paper? Thank you very much again.

We thank the reviewer for these valuable comments and suggestions.

As the reviewer suggested, we added descriptions of the data with synaptic blockers and the assumption in the Discussion section **(Page 16, Lines 377–386)**. We also added descriptions of the firing patterns of PCs while citing the paper **(Pages 7–8, Lines 169–176)**.
